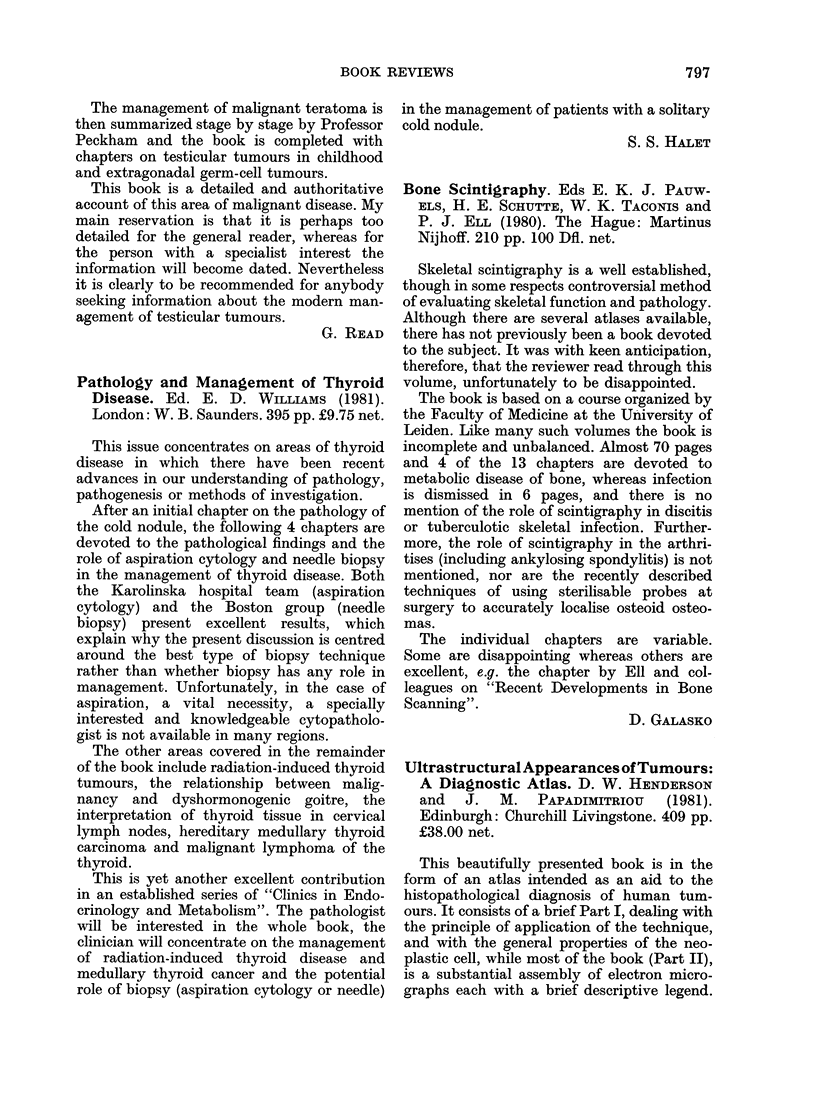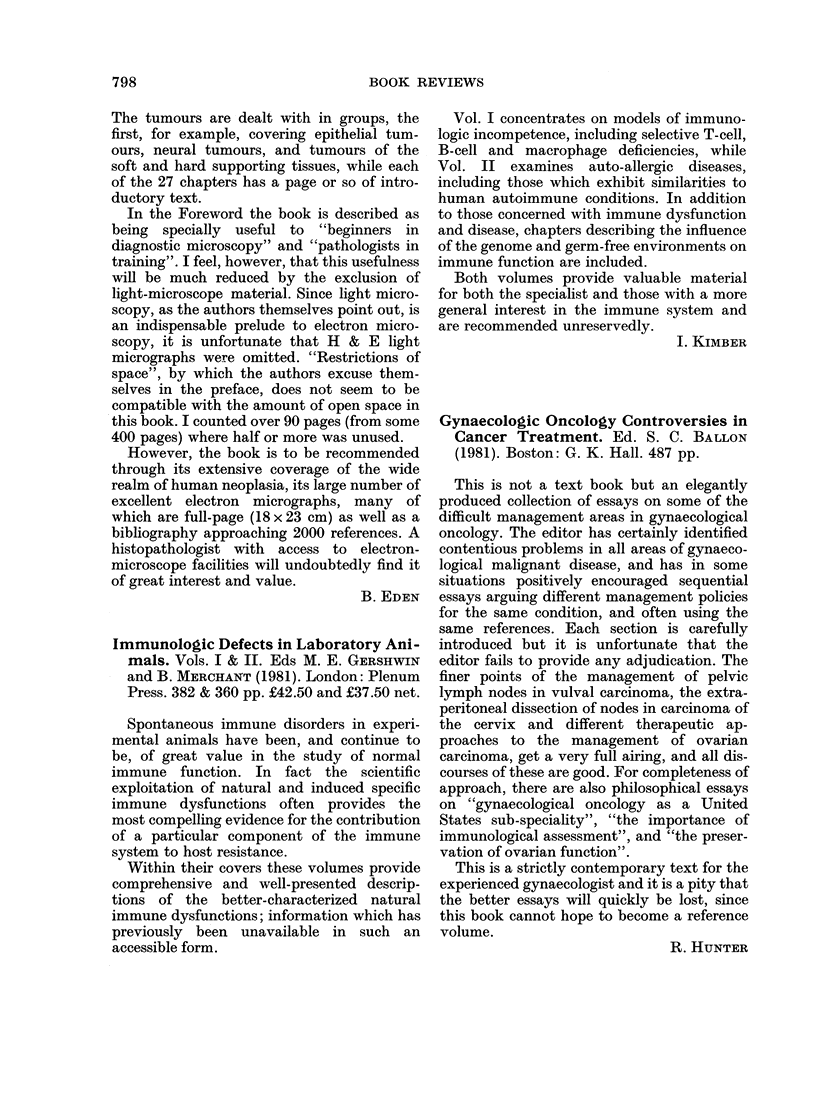# Ultrastructural Appearances of Tumours: A Diagnostic Atlas

**Published:** 1982-05

**Authors:** B. Eden


**Ultrastructural Appearances of Tumours: A Diagnostic Atlas.** D. W. HENDERSON and J. M. PAPADIMITRIOU (1981). Edinburgh: Churchill Livingstone. 409 pp. £38.00 net.

This beautifully presented book is in the form of an atlas intended as an aid to the histopathological diagnosis of human tumours. It consists of a brief Part I, dealing with the principle of application of the technique, and with the general properties of the neoplastic cell, while most of the book (Part II), is a substantial assembly of electron micrographs each with a brief descriptive legend.

The tumours are dealt with in groups, the first, for example, covering epithelial tumours, neural tumours, and tumours of the soft and hard supporting tissues, while each of the 27 chapters has a page or so of introductory text.

In the Foreword the book is described as being specially useful to "beginners in diagnostic microscopy" and "pathologists in training". I feel, however, that this usefulness will be much reduced by the exclusion of light-microscope material. Since light microscopy, as the authors themselves point out, is an indispensable prelude to electron microscopy, it is unfortunate that H & E light micrographs were omitted. "Restrictions of space", by which the authors excuse themselves in the preface, does not seem to be compatible with the amount of open space in this book. I counted over 90 pages (from some 400 pages) where half or more was unused.

However, the book is to be recommended through its extensive coverage of the wide realm of human neoplasia, its large number of excellent electron micrographs, many of which are full-page (18 x 23 cm) as well as a bibliography approaching 2000 references. A histopathologist with access to electron-microscope facilities will undoubtedly find it of great interest and value.

B. Eden